# Differentiation of *Candida albicans* Species Complex by Tobacco Agar Obtained from Different Cigarette Brands Available in Colombia

**DOI:** 10.1155/2020/5438967

**Published:** 2020-03-21

**Authors:** Soraya E. Morales-López, Jayr Yepes, Danna C. Elles, Lisahidy Macías, Abid Cañate, Jorge Robles Camargo

**Affiliations:** ^1^Grupo CINBIOS, Departamento de Microbiología, Universidad Popular del Cesar, 200004 Valledupar, Colombia; ^2^Grupo BIOTECGEN, Departamento de Microbiología, Universidad Popular del Cesar, 200004 Valledupar, Colombia; ^3^Grupo de Investigación Fitoquímica Universidad Javeriana-GIFUJ, Pontificia Universidad Javeriana, 110231 Bogotá, Colombia

## Abstract

**Aim:**

To evaluate the efficacy of different brands of cigarettes in the preparation of tobacco agar, for the differentiation of these related yeasts. *Methodology*. Tobacco agar was prepared using six brands and four varieties of cigarettes, and 125 clinical isolates previously identified by PCR and Maldi-Tof were used. To determine whether the results of the microbiological tests were associated with similarities in the chemical components of cigarettes, thin-layer chromatography was performed.

**Results:**

*Candida dubliniensis* colonies presented hue differences according to the incubation temperature and the brand or variety of cigarette used, except in the tobacco agar produced with Marlboro Xpress cigarette, where its differentiation was not possible. The chromatograms showed few differences among apolar and medium polarity extract components.

**Conclusions:**

Tobacco agar is a low-cost tool used for the differentiation of *Candida dubliniensis*; however, incubation temperature and cigarette brand affect the performance of the media. No relationship was found between the microbiological results and the chemical similarity of the extracts of the cigarettes by chromatography.

## 1. Introduction

*Candida albicans* is the main human fungal pathogen; however, *Candida dubliniensis* and *Candida africana* (a biovariety of *C. albicans)* are emerging yeasts gaining clinical and epidemiological relevance that share morphological and physiological characteristics, thus forming the *Candida albicans* complex [1, 2].

Due to the phenotypic similarities, these isolates can be identified as *C. albicans*, causing an overestimation in their epidemiology [3]. Although several phenotypic identification systems are designed to differentiate *C. dubliniensis* from *C. albicans*, they do not allow identifying *Candida africana* [4]. The discrimination between these species is relevant for the understanding of their clinical importance and epidemiological role in human infections [5–7].

The tobacco agar allows, in most cases, the differentiation between *C. dubliniensis* and *C. albicans*; but it is not used in Colombia as a routine medium. In addition, there are no reports of their use for *C. africana* isolates.

The aim of this work is to evaluate the effectiveness of cigarettes available in Colombia for the preparation of tobacco agar on the differentiation of *C. albicans*, *C. dubliniensis*, and *C. africana.*

## 2. Materials and Methods

### 2.1. Strains

Clinical isolates of *C. albicans* (100), *C. dublinienisis* (20), and *C. africana* (5) were used. Isolates of *C. albicans* were obtained from the Culture Collection of the Microbiology Laboratory of Universidad Popular del Cesar (Valledupar, Colombia), whereas isolates of *C. dubliniensis* and *C. africana* were donated by the mycology laboratories of Instituto Nacional de Salud (INS, Colombia), the Laboratory of Proteomics and Human Mycoses of Pontificia Universidad Javeriana (PUJ), and the Laboratory of Mycology and Molecular Diagnostics of Universidad Nacional del Litoral (UNL, Argentina). All isolates were previously identified by MALDI-TOF and PCR.

### 2.2. Culture Media Preparation

12.5 g of each cigarette of national distribution was weighed: Rhotmans, Fortuna, Marlboro (Classic, Fussion, Gold, Xpress varieties), Lucky Strike, L & M, and Chesterfield; then, they were added to respective Erlenmeyer flasks with 250 mL of distilled water. The mixes were boiled for 30 minutes in a water bath and then filtered with cheesecloth to obtain the extracts, which were then diluted to 250 mL with distilled water. 5 g of agar was added to the extracts and sterilized at 121°C for 15 minutes. Finally, each medium was cooled to 55°C and poured into 90 mm petri dishes.

### 2.3. Culture

Fresh cultures on the Sabouraud agar (peptone 1%, glucose 2%, and agar 2%) were used and then streaked on tobacco agar plates of each cigarette. Plates were incubated at 37°C and 28°C for 7 days, separately. Variations of macromorphology (colony color and margin) and micromorphology (presence of chlamydoconidia at 100x and 400x magnification when the microorganism was stained with lactophenol cotton blue) were registered daily. The experiment was carried out in triplicate. *C. albicans* ATCC 28367, *C. albicans* ATCC 90028, and *C. dubliniensis* ATCC 3949 and ATCC 11473 were used as control strains.

### 2.4. Thin-Layer Chromatography

This technique was performed to determine commonalities between the microbiological results and the TLC banding patterns of the extracts. The cigarettes were randomly numbered as follows: (1) Fortune, (2) Rhotmans, (3) L & M, (4) Marlboro Classic, (5) Marlboro fusion, (6) Marlboro gold, (7) Marlboro Xpress, (8) Lucky Strike, (9) Chesterfield, and (10) Philip Morris (of Argentine origin). 3 g of each variety of cigarette was subjected to consecutive extraction with petroleum ether, dichloromethane, and ethanol by cold maceration in an orbital shaker at 114 rpm for 120 hours [8]. The extracts were concentrated by using a rotavapor (Buchi R-111) at a maximum temperature of 40°C. Later, the residual solvent was brought to dryness in a vacuum oven at 30°C. The most appropriate mobile phase was selected by performing preliminary thin-layer chromatography in which the dried extracts were redissolved and then eluted on Silica Gel 60 G with fluorescence marker F254 as the stationary phase. The petroleum ether, dichloromethane, and ethanol extracts were eluted with petroleum ether : acetone (8 : 2), toluene : acetone (8 : 2), and three proportions of dichloromethane : ethanol (8 : 2, 7 : 3 and 6 : 4), respectively. Long-wave ultraviolet (UV) light (365 nm), short-wave UV light (254 nm), and 5% (w/v) vanillin-sulfuric acid were used as visualization agents [9].

## 3. Results

In all media and at both incubation temperatures, *C. albicans* and *C. albicans* var. africana produced smooth-edged, white-to-cream color colonies, without chlamydoconidia and without or with poorly developed pseudomycelium. No variations were observed when extending the incubation up to 10 days.

**Figure 1:**
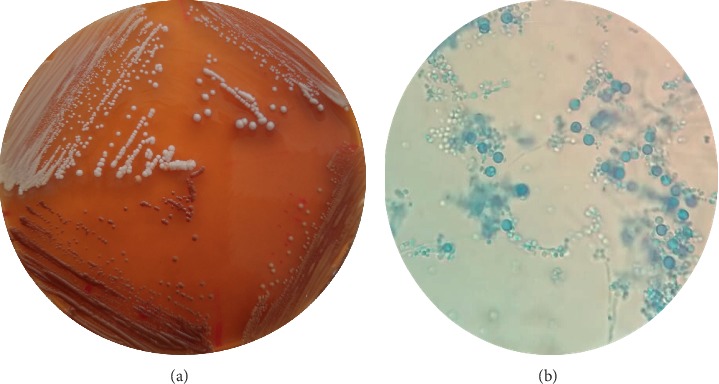
(a) *C. dubliniensis* (bottom) produced yellowish-brown colonies (darker color) on tobacco agar prepared from Marlboro Classic incubated at 25°C, while *C. albicans* (top left) and *C. africana* (top right) produced white-to-cream-colored colonies. (b) Lactophenol cotton blue mount of *C. dubliniensis* from Marlboro Classic tobacco agar showing large and spherical chlamydoconidia (400X).

Colonies of *C. dubliniensis* displayed differences in hue, depending on the incubation temperature and the brand of cigarette used; at 37°C, yellowish brown colonies (darker color) were observed on agar media prepared with Fortuna and Marlboro Classic cigarettes (Figure 1(a)); yellowish brown color colonies (pale color) on media prepared with Marlboro Fusion, Marlboro Gold, and Rhotmans cigarettes; and white and smooth colonies in media prepared with Marlboro Xpress, L & M, Chesterfield, and Lucky Strike cigarettes.

At 25°C, *C. dubliniensis* produced yellowish brown colonies (darker color) on tobacco agar prepared from Marlboro Classic, Marlboro Gold, Fortuner, and Marlboro Fussion cigarettes and colonies with pale color (weak positive) in tobacco agar prepared from Rhotmans, Lucky Strike, L & M, and Chesterfield cigarettes. No differences were observed between the colony color of *C. dubliniensis*, *C. albicans* and *C. africana* on the agar prepared with Marlboro Xpress cigarettes. On agar media prepared from Phillip Morris cigarettes (external control), *C. dubliniensis* produced intense yellowish-brown colonies. Remarkably, in all media, *C. dubliniensis* produced scalloped margin colonies (Table 1).

Microscopic examination of *C. dubliniensis* growing on Marlboro Classic, Marlboro Gold, and Marlboro Fussion revealed abundant production of large and spherical chlamydoconidia (Figure 1(b)), whereas on tobacco agar prepared from Fortuna cigarettes, only few small and subspherical chlamydoconidia were detected.

**Figure 2:**
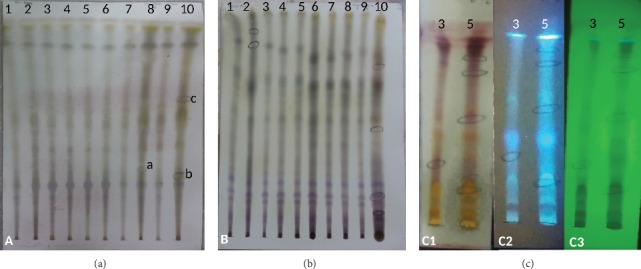
Chromatograms using different solvents as a mobile phase. (a) Petroleum ether : acetone (8 : 2) revealed with vanillin: spots a (Rf = 0.31), b (Rf = 0.28), and c (Rf = 0.65) indicate the presence of mono- or sesquiterpenes; (b) toluene : acetone (8 : 2) revealed with vanillin: samples 2, 6, 7, 8, and 10 have a common spot; (c) chromatograms of samples number 3 and 5 obtained with dichloromethane : ethanol in an 8 : 2 ratio, revealed with (C1) vanillin, (C2) long-wave UV light (365 nm), and (C3) short-wave UV light (254 nm).

The chromatogram obtained with petroleum ether : acetone (8 : 2) and revealed with vanillin had differences in the spots of the Lucky Strike cigarette samples (8) (A, with Rf = 0.31) and 10 (B, with Rf = 0.28 and C, with Rf = 0.65), as well as a higher concentration in some bands (Figure 2(a)). In the other samples, similar migrations of the bands were observed. With short wave UV light, the same migration bands were observed in all the samples, while with long UV light, the same bands were observed, but with a higher concentration in samples 8 and 10 (Lucky Strike and Phillip Morris).

Concentrated spots were observed in samples 2, 6, 7, 8, and 10 on TLC plates eluted with toluene : acetone (8 : 2) and revealed with vanillin. In sample 2 (Rhotmans), two additional spots of lower polarity were observed, and the sample 10 (Phillip Morris) presented 3 spots of different polarities, which were not found in the other samples. The bands of less migration were similar in all the samples (Figure 2(b)).

With short-wave UV light, two spots were observed in sample 2 and one spot in sample 10, which did not appear after the revelation with vanillin. In addition, some bands had slight differences between the samples. In the visualization with long UV light, the spots of samples 5, 6, 7, 8, 9, and 10 showed a higher concentration. Furthermore, some of the bands of less migration were similar in all the samples.

Additional TLC of the ethanol extract samples 3 and 5 (which showed differences with the rest) was performed. The plates were eluted with three proportions of dichloromethane : ethanol (8 : 2, 7 : 3, and 6 : 4). Finally, a predominance of yellow and red bands was observed with vanillin reagent, an indicative of flavonoid and terpene compounds (Figure 2(c)).

## 4. Discussion

In this study, all *C. dubliniensis* produced brown or yellowish colonies with chlamydoconidia in most media, and all *C. albicans* produced smooth white-to-cream colonies without chlamydoconidia. These results are similar to those described by Pineda et al. in Argentina, who used Exceter ^®^ pipe tobacco and 100% *C. dubliniensis* isolates produced brownish and rough colonies, whereas 98.5% of *C. albicans* isolates produced white and smooth colonies. Incubation was mantained for 48–72 hours at 30°C (*n* = 212 isolates) [10].

In 2011, Bosco-Borgeat et al. (Argentina) used tobacco agar for presumptive identification of *C. dubliniensis* (sensitivity of 80% and specificity of 100%), employing Phillip Morris® cigarettes and on tobacco agar, 5/183 *C. albicans* produced scarce and solitary chlamydoconidia and all the *C. dubliniensis* isolates produced abundant chlamydoconidia in clusters. Moreover, 8/10 *C. dubliniensis* produced rough and yellowish colonies, and all (183) *C. albicans* isolates produced smooth and white-to-cream-colored colonies [11].

In Brazil, Silveira-Gomes et al. used tobacco from Fumo Extra Forte®, and the culture plates were incubated at 28°C and observed daily up to 96 h. Morphological aspects suggestive of *C. dubliniensis* on tobacco agar (rough/brownish-yellow colonies and hyphae with chlamydoconidia) were observed in nine isolates [12]; Monteiro included 200 isolates, presumptively identified as *C. albicans* or *C. dubliniensis*, that were inoculated on tobacco agar and incubated at 28°C for 4 days. However, all the isolates were further identified as *C. albicans* by PCR [13].

Recently, Oliveira-Liveiro (Brazil, 2017) reported a sensitivity of 81.5%, when comparing the phenotypic results of *C. albicans* and *C. dubliniensis*, with the results of molecular techniques. In the study, six strains produced brownish and rough colonies and developed chlamydoconidia, but only two were identified as *C. dubliniensis* by PCR. Furthermore, one of the isolates, identified as *C. dubliniensis* by PCR, did not produce chlamydoconidia on tobacco agar [7].

In our study, *C. dubliniensis* macroscopic differences were subtle or absent in the incubation at 37°C, so that we suggest the incubation at 28°C on agar media prepared from Marlboro Classic, Marlboro Gold, or Marlboro Fussion cigarettes. Additionally, it was not possible to discriminate *C. dubliniensis* on tobacco agar produced from Marlboro Xpress cigarettes, and no agar allowed the differentiation between *C. albicans* and *C. albicans var. africana.*

After an exhaustive bibliographic search for the literature in English and Spanish, using the electronic database Medline and LILACS and the terms “cigarette,” “tobacco agar,” and “*Candida*,” it was not possible to find a study comparing different brands of cigarettes for the preparation of tobacco agar, nor with the use of thin-layer chromatography. However, when the search was performed for the gray literature in Google and Google scholar, only one document was found comparing the discrimination of *C. dubliniensis* from *C. albicans* using three brands of local cigarettes in Perú; no differences were found according the cigarette brand [14].

The extracts of cigarettes analyzed by TLC were similar to each other regarding apolar and medium polarity components (simple phenols, monoterpenes, sesquiterpenes, and diterpenes), according to the color of the blue spots revealed with long UV light and with vanillin. In extracts of medium polarity, the spots of all the samples probably represent some alkaloids and triterpene molecules.

The presence of a small compound, similar to formic acid, was also detected. The toluene : acetone fraction corresponds to medium polarity, which means that these compounds show variation in all 10 cigarette samples.

In the polar (ethanolic) extract, blue fluorescent bands were present under long-wave UV light. These bands are characteristic of complex phenols like flavonoids, anthocyanins, and tannins and some derivatives of quinones and anthraquinones.

## 5. Conclusions

In conclusion, the tobacco agar described by Tendolkar et al. for the differentiation of *Cryptococcus neoformans,* [15] and later by Khan et al. for the differentiation of *C. dubliniensis* from *C. albicans*, [16] varies in capacity of discrimination of *C. dubliniensis* according to the brand of cigarette used and the incubation temperature. Interestingly, no relationship was found between the microbiological results and the TLC band patterns of the extracts; therefore, an exhaustive analysis of the chemical composition and its relationship with the performance of tobacco agar can be achieved with gas chromatography and mass spectrometry.

## Figures and Tables

**Table 1:** Macroscopic (color and margin of the colony) and microscopic (frequency, size, and shape of the chlamydoconidia) characteristics of *C. dubliniensis* according to the cigarette used for the tobacco agar preparation.

Cigarette brand	Incubation at 37°C	Incubation at 25°C	Colony margin	Chlamydoconidia
Fortuna	+	+	Sc	SSSp
Marlboro classic	+	+	Sc	ALS
Marlboro fusion	+^*p*^	+	Sc	ALS
Marlboro gold	+^*p*^	+	Sc	ALS
Rhotmans	+^*p*^	+^*d*^	Sc	SSSp
Marlboro express	−	−	E	SSSp
L & M	−	+^*d*^	Sc	SSSp
Chesterfield	−	+^*d*^	Sc	SSSp
Lucky strike	+	+^*d*^	Sc	SSSp

+: dark brown; +*^p^*: pale brown; −: yellow; Sc: scalloped; E: entire; SSSp: scarce, small, and subspherical; ALS: abundant, large, and spherical.

## Data Availability

The data used to support the findings of this study are available from the corresponding author upon request.
